# Bridging classical and quantum mechanics

**DOI:** 10.1088/0026-1394/53/5/a83

**Published:** 2016

**Authors:** D Haddad, F Seifert, L S Chao, S Li, D B Newell, J R Pratt, C Williams, S Schlamminger

**Affiliations:** 1 National Institute of Standards and Technology (NIST), 100 Bureau Drive Stop 8171, Gaithersburg, MD 20899, USA; 2 University of Maryland, Joint Quantum Institute, College Park, MD 20742, USA; 3 Department of Electrical Engineering, Tsinghua University, Beijing 100084, People’s Republic of China

**Keywords:** watt balance, frequency comb, Josephson voltage, quantum Hall resistance, mass-energy equivalence

## Abstract

Using a watt balance and a frequency comb, a mass-energy equivalence is derived. The watt balance compares mechanical power measured in terms of the meter, the second, and the kilogram to electrical power measured in terms of the volt and the ohm. A direct link between mechanical action and the Planck constant is established by the practical realization of the electrical units derived from the Josephson and the quantum Hall effects. By using frequency combs to measure velocities and acceleration of gravity, the unit of mass can be realized from a set of three defining constants: the Planck constant *h*, the speed of light *c*, and the hyperfine splitting frequency of ^133^Cs.

## 1. Watt balances in classical mechanics

Forty years ago, Bryan Kibble [1] published a fundamental insight that significantly changed the field of fundamental electrical metrology, and later mass metrology: the coupling factor between force and current in a motor is the same as the one between voltage and velocity in a generator configuration. The watt balance experiment was born. In this experiment, mechanical balance is used to compare an electromagnetic force produced by a coil in a magnetic field to the weight of a test mass, i.e.

(1) $$F_{z}=mg=-I\frac{\partial \Phi }{\partial z},$$

where *m* is the mass of a test mass, *g* the local gravitational acceleration, *I* the current in the coil, and Φ the magnetic flux linkage to the coil. Before 1976, the calculation of the flux from this coil, which is the integral of the magnetic flux density ***B*** over the area segment d***A***, required absolute measurements of the coil physical dimensions, which is difficult and limits the precision that could be achieved. Kibble proposed to determine the vertical derivative of the magnetic flux from the quotient of the induced voltage *U* to the vertical velocity *v*_*z*_ when the coil is moved vertically in the same magnetic field,

(2) $$-\frac{\partial \Phi }{\partial z}=\frac{U}{v_{z}}.$$

**Figure 1.**
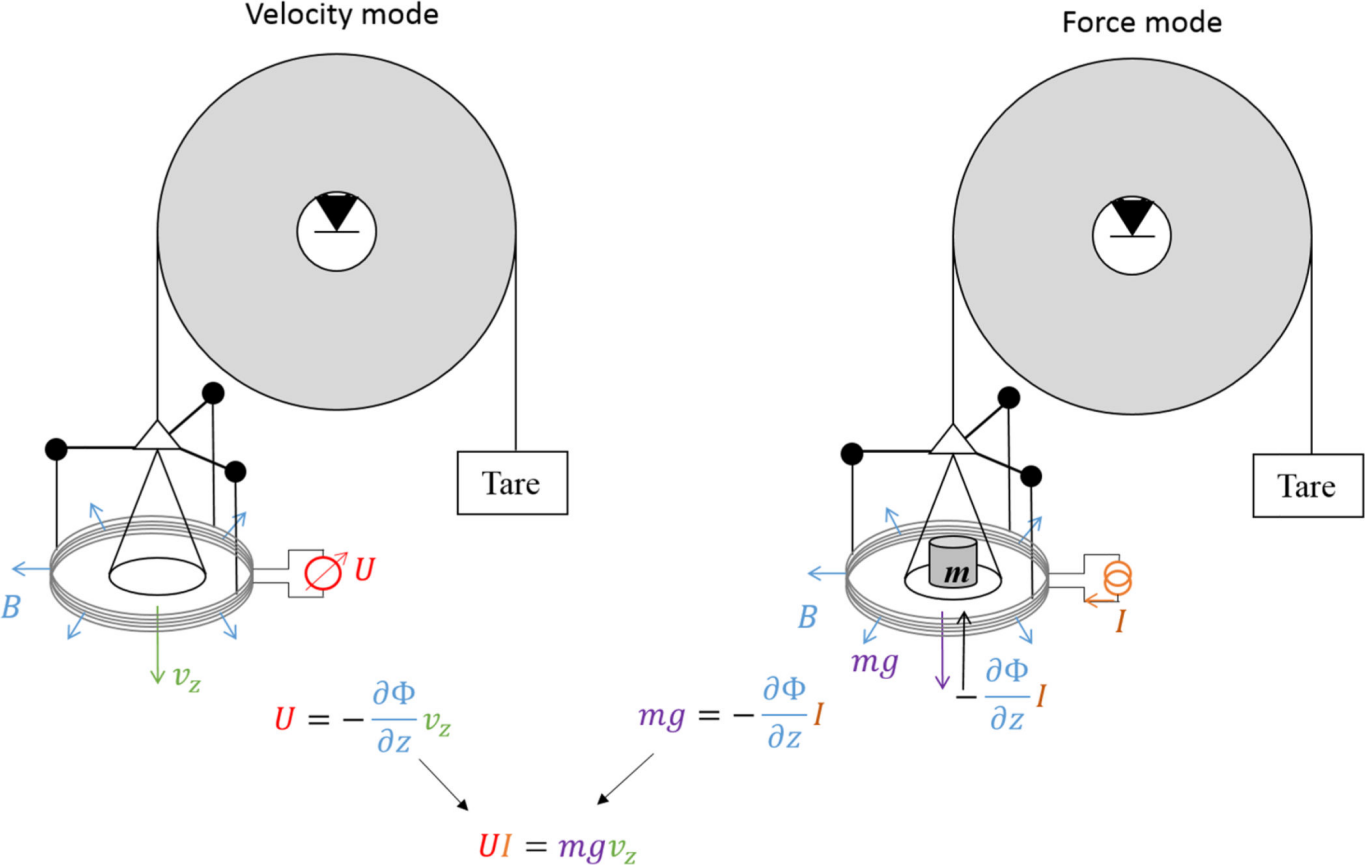
The principle of a watt balance using a pulley. In velocity mode (left), a drive force rotates the pulley and hence the coil moves vertically through the magnetic field. An induced voltage *U* across the coil is generated which is proportional to the product of the vertical velocity *v*_*z*_, and the negative derivative of the flux with respect to *z*, i.e. −*∂*Φ/*∂z*. By measuring *U* and *v*_*z*_, *∂*Φ/*∂z* can be obtained by a simple division. In the force mode (right), the electromagnetic force −*I∂*Φ/*∂z* is generated by the coil carrying current *I* placed in the same magnetic field. The current *I* in the coil is adjusted to maintain the position of the pulley to a nominal position.

The derivative of the flux with respect to *z* is eliminated by combining equations (1) and (2) together leading to

(3) $$mgv_{z}=UI,$$

which describes an equivalence between virtual mechanical and electrical power. The power is virtual because voltage and current, as well as weight and velocity, are measured in two separate phases. The principle of the watt balance experiment is presented in the simplified scheme of figure 1.

## 2. The quantum leap

In 1962, Brian Josephson [2] predicted that if a junction, consisting of a thin insulating barrier and two superconductors on either side, is driven at a frequency *f*, then the current voltage curve (*I* − *V*) will develop regions of constant voltage. This became the basis of voltage standards starting in 1970. The potential difference between the two superconductors in the presence of a bias current is given by $K_{\text{J}}^{-1}f$. Here *K*_J_ denotes the Josephson constant given by *K*_J_ = 2*e/h*, where *e* is the elementary charge and *h* the Planck constant. By using *n* junctions in series, a larger voltage $U=nK_{\text{J}}^{-1}f$ can be obtained.

The quantum Hall effect occurs in a sample that confines electric current to two dimensions at sufficiently low temperatures. If such a sample is exposed to a high magnetic field, the quotient of the transverse voltage to the current flowing in the device, is quantized. This quantum Hall resistance is equal to an integer fraction of *R*_K_ = *h/e*^2^. The constant *R*_K_ is named after von Klitzing to honor his discovery of this effect in 1980 [3]. Quantum Hall resistance standards of value *R* are realized in terms of the von Klitzing constant $R=R_{K}/p$, where *p* is an integer.

In the watt balance experiment, the weighing current needs to be determined accurately. This is done, by passing the current *I* through a resistor with resistance *R* and measuring the potential difference *U* across this resistor. Equation (3) can now be expressed as

(4) $$mgv_{z}=\frac{U^{2}}{R}.$$

To simplify the equations and without loss of generality, the resistance value *R* is chosen such that the potential difference across it is equal to the induced voltage. However, this is not necessary for the reasoning outlined below.

Replacing *U* and *R* in equation (4) with the expressions from the Josephson effect (twice) and the quantum Hall effect yields

(5) $$mgv_{z}=\frac{pn^{2}}{4}f^{2}h.$$

Conveniently, the elementary charge cancels out in the product $K_{\text{J}}^{2}R_{\text{K}}=4/h$. A relation between a macroscopic mass and the Planck constant is established. A paper [6] to be published in the same issue presents a more complete overview on watt balance experiments.

## 3. The unit of mass from the Planck constant

In the International System of Units (SI), the unit of mass, the kilogram, is still defined by an artifact, the international prototype of the kilogram (IPK) rather than a fundamental constant of nature. The present SI definition of the unit of mass is: ‘the kilogram is the unit of mass; it is equal to the mass of the international prototype of the kilogram’. Plans are under way to redefine the SI in 2018, for a more fundamental system of units not based on artifacts. In the revised SI, the plan for the unit of mass is that it can be realized using a watt balance from the fixed value of the Planck constant *h* [4, 5] by rewriting equation (5) as

(6) $$m=\frac{pn^{2}}{4}\frac{f^{2}}{gv_{z}}h,$$

where all quantities are based on their determined values in SI units. A paper [6] to be published in the same issue presents a comprehensive overview on realizing the unit of mass using watt balance experiments.

## 4. Frequency, the last piece of the puzzle

The second will be the most accurately realized unit in the revised SI. Hence it will be advantageous to derive quantities in equation (6) from frequency measurements.

The frequency *f* can be written as a rational multiple, *k*_*u*_, of the hyperfine splitting frequency of ^133^Cs,

(7) $$f=k_{u}\Delta \nu {(}^{133}\text{Cs)hfs}.$$

In watt balance equations, the velocity can be expressed as a rational multiple *k*_*v*_ of the speed of light

(8) $$v_{z}=\frac{s_{v}}{t_{v}}=k_{s}\lambda f_{v}=k_{s}\frac{f_{v}}{f_{o}}c=k_{v}c.$$

The ratio of the radio frequency *f*_*v*_ to the optical frequency *f*_*o*_ can be determined very accurately with the development of the optical frequency combs [7], which can act as ‘clock gears’.

Similarly, with a frequency comb, the local acceleration can be written as a product of a constant *k*_*g*_, the speed of light and the hyperfine splitting frequency of ^133^Cs,

(9) $$g=\frac{v_{g}}{t_{g}}=k_{vg}ck_{ug}^{133}\text{Cs}=k_{g}c\Delta \nu {(}^{133}\text{Cs)hfs}.$$

Replacing the quantities *g*, *v*_*z*_ and *f*_*U*_ in equation (6) with the expressions from equations (7)–(9) yields

(10) $$m=\frac{pn^{2}}{4}\frac{k_{u}^{2}}{k_{g}k_{v}}\frac{h\Delta \nu {(}^{133}\text{Cs)hfs}}{c^{2}}.$$

The constants can be summarized into one, i.e. $k_{m}=pn^{2}k_{U}^{2}/\left(4k_{g}k_{v}\right)$,which allows one to write the above equation into a form resembling the mass-energy equivalence,

(11) $$mc^{2}=k_{m}h\Delta \nu {(}^{133}\text{Cs)hfs}.$$

The macroscopic test mass *m* is written as a multiple of the relativistic photon mass corresponding to the energy difference of the two hyperfine levels in the Cesium atom, i.e. approximately 6.777 × 10^−41^ kg. Although the mass-energy equation has been theoretically recognized in numerous papers [8–10] as a possible route to redefine the unit of mass, this short note presents the practical experimental means to calibrate the factor *k*_*m*_. In summary, by measuring the velocity and the gravitational acceleration in the watt balance experiment with frequency combs, the unit of mass can be realized from a set of three defining constants: the Planck constant *h*, the speed of light *c* and the hyperfine splitting frequency of ^133^Cs.
